# Characterization of the CCAAT-binding transcription factor complex in the plant pathogenic fungus *Fusarium graminearum*

**DOI:** 10.1038/s41598-020-61885-4

**Published:** 2020-03-17

**Authors:** Jung-Eun Kim, Hyejin Nam, Jiyeun Park, Gyung Ja Choi, Yin-Won Lee, Hokyoung Son

**Affiliations:** 1 Research Institute of Agriculture and Life Sciences and Department of Agricultural Biotechnology, Seoul National University, 08826 Seoul, Republic of Korea; 2 Therapeutic & Biotechnology Division, Center for Eco-friendly New Materials, Korea Research Institute of Chemical Technology, Daejeon, 34114 Republic of Korea

**Keywords:** Fungal genetics, Fungal pathogenesis

## Abstract

The CCAAT sequence is a ubiquitous *cis*-element of eukaryotic promoters, and genes containing CCAAT sequences have been shown to be activated by the CCAAT-binding transcription factor complex in several eukaryotic model organisms. In general, CCAAT-binding transcription factors form heterodimers or heterotrimeric complexes that bind to CCAAT sequences within the promoters of target genes and regulate various cellular processes. To date, except Hap complex, CCAAT-binding complex has been rarely reported in fungi. In this study, we characterized two CCAAT-binding transcription factors (Fct1 and Fct2) in the plant pathogenic fungus *Fusarium graminearum*. Previously, *FCT1* and *FCT2* were shown to be related to DNA damage response among eight CCAAT-binding transcription factors in *F. graminearum*. We demonstrate that the nuclear CCAAT-binding complex of *F. graminearum* has important functions in various fungal developmental processes, not just DNA damage response but virulence and mycotoxin production. Moreover, the results of biochemical and genetic analyses revealed that Fct1 and Fct2 may form a complex and play distinct roles among the eight CCAAT-binding transcription factors encoded by *F. graminearum*. To the best of our knowledge, the results of this study represent a substantial advancement in our understanding of the molecular mechanisms underlying the functions of CCAAT-binding factors in eukaryotes.

## Introduction

Gene expression is primarily orchestrated by a set of transcription factors that bind to *cis*-elements in promoter regions^1^. In addition to the TATA-box, the CCAAT sequence is a ubiquitous *cis*-element of eukaryotic promoters that is present in the promoters of approximately 30% of eukaryotic genes^2^. Therefore, genes carrying a CCAAT-box are known to be primarily activated by conserved CCAAT-binding complexes in model eukaryotic organisms^3^.

The CCAAT-binding complex (CBC) typically consists of heterotrimeric core subunits and regulates primary/secondary metabolism, development, stress responses, and virulence in animals, plants, and fungi^4–7^. The heme activator protein (HAP) complex, which is also termed nuclear factor Y (NF-Y) or CCAAT-binding factor (CBF), was the first identified and is the most well studied CBC in various eukaryotic organisms. The *Saccharomyces cerevisiae* Hap complex consists of three essential CCAAT-binding factors (Hap2p, Hap3p, and Hap5p) that are indispensable for CCAAT-binding activity^8,9^, orthologues of which (NF-YA/CBF-B, NF-YB/CBF-A, and NF-YC/CBF-C, respectively) also comprise the mammalian CCAAT complex (NF-Y/CBF)^10^. Core elements of Hap3 and Hap5 display amino acid sequence similarities to the histone fold motifs of histones H2B and H2A, respectively, which are responsible for heterodimeric interactions. Another essential element of the complex, Hap2, contains a subunit association domain that allows for heterotrimer formation and nuclear localization signals (NLS)^11,12^. After the assembly of Hap2, Hap3, and Hap5, the heterotrimeric complex then recruits Hap4, an additional component that is only present in fungi, which allows for subsequent binding to the promoter of the target genes containing the CCAAT sequence^3,12^.

Similar to Hap5 and Hap3, two small components of DNA polymerase epsilon (Pol ε), DNA polymerase II subunit B3 (Dpb3) and Dpb4 are known that harbour H2A/H2B-like histone fold motifs in *S*. *cerevisiae* and *Schizosaccharomyces pombe*, as well as in plants and humans^13–16^. Pol ε plays crucial roles in chromosome replication, cell cycling, and the repair of damaged DNA^17^. Both Dpb3 and Dpb4 are non-essential proteins that form a heterodimeric complex and bind to double-stranded DNA^18–20^. The Dpb3-Dpb4 complex physically associates with the Pol ε catalytic subunit, Cdc20, and also interacts with proteins that are important for heterochromatin assembly. However, orthologues of Dpb3 and Dpb4 have rarely been identified in filamentous fungi to date.

The HAP complex is responsible for global transcriptional activation, and several genes directly regulated by the HAP complex have been characterized in filamentous fungi. The HAP complex of *Aspergillus nidulans* (AnCF) is involved in the utilization of carbon and nitrogen sources, and several enzyme-coding genes (e.g., *amdS*, *taaG2*, and *ipnA*) are positively regulated by AnCF^3,21^. The *Aspergillus oryzae* HAP complex, AoCP, is a direct activator of the Taka-amylase A gene (*taa*)^22^. In addition, activation of the gene encoding the cellobiohydrolase II cellulose degrading enzyme (*cbh2*) is mediated by the Hap3 orthologue (HapC) in the biomass-degrading fungus *Trichoderma reesei*^23^. Recently, a few orthologues of HAP complex components and the histone-like protein have been characterized in plant pathogenic fungi, including *Fusarium* species^24–26^. These proteins are involved in pathogenesis, fungal development, and various biological processes, yet interactions between CCAAT-binding factors have not been investigated in plant pathogenic fungi, including *F. graminearum*.

The ascomycete fungus *F. graminearum* is a prominent plant pathogen that causes Fusarium head blight (FHB) in cereal crops and ear and stalk rot on maize^27^, all of which result in severe yield losses and an accumulation of mycotoxins (e.g., trichothecenes and zearalenone) that are harmful to animals and humans^28^. Previously, we performed a genome-wide functional analysis of the complete repertoire of transcription factor-encoding genes in *F. graminearum*^29^, which resulted in the identification of eight transcription factors containing CCAAT-binding domains^29^. Interestingly, while the phenotypes of *F. graminearum* strains mutated for six out of eight genes were not significantly different from those of the wild-type strain, the deletion of two putative CCAAT-binding transcription factors resulted in defects in fungal development, including increased sensitivity to DNA damaging agents^29,30^. Furthermore, a recent study revealed that the expression of *FgHLTF1*, one of the two CCAAT-binding factors involved in the DNA damage response, was downregulated by the putative type 2A phosphatase FgPpg1 and was shown to be associated with the high osmolarity glycerol (HOG) pathway^24^.

In this study, we attempted to characterize the CCAAT complex structure and its biological functions in *F. graminearum*. Our results demonstrate that *F. graminearum* has two distinct CCAAT complex components containing histone-fold motifs, Fct1 and Fct2 (FgHltf1), which are required for DNA damage responses, sexual development, virulence, and trichothecene production. A protein-binding microarray analysis revealed that Fct2 binds to the consensus sequence CCAAT, and we also confirmed that Fct1 and Fct2 interact to form a complex. Moreover, this study provides the strong evidence supporting that the CCAAT-binding complex only has two distinct CCAAT-binding factors in *F. graminearum*.

## Results

### Identification and cellular localization of CCAAT-binding factors

In our previous study, we identified 16 transcription factors involved in DNA damage responses in *F. graminearum*^30^. Among them, strains carrying mutations in two CCAAT-binding factor-encoding genes, *GzCCAAT002* (FGSG_01182) and *GzCCAAT004* (FGSG_05304), were highly sensitive to DNA damaging agents compared to the wild-type strain. Recently, *GzCCAAT004* was identified as a putative histone-like transcription factor, *FgHLTF1*, through a transcriptome analysis of a Δ*Fgppg1* strain^24^. In this study, we designated *GzCCAAT002* and *GzCCAAT004* as *F*. *graminearum* CCAAT-binding transcription factor 1 (*FCT1*) and *FCT2*, respectively. To generate complementation strains, the geneticin resistance gene cassette^31^ in each deletion mutant was replaced with *FCT1* or *FCT2* fused to the green fluorescent protein-encoding gene (*GFP*) and a hygromycin resistance gene cassette (*HYG*), yielding the strains FCT1c and FCT2c (Table 1 and Supplementary Fig. S1).

**Table 1:** *F. graminearum* strains used in this study.

Strain	Genotype	Source or reference
Z-3639	Wild-type *Fusarium graminearum*	^53^
HK12	*GFP-HYG* (GFP constitutive expresser in cytosol)	^63^
KM19	Δ*mat1-1-1::GEN; GFP-HYG*	^34^
mat1r	∆*mat1-1-1::GEN; hH1::hH1-RFP-GEN*	^55^
*fct1*	∆*fct1::GEN*	∆*gzccaat02*^29^
*fct2*	∆*fct2::GEN*	∆*gzccaat04*^29^
*fct1 fct2*	∆*fct1::GEN;* ∆*fct2::HYG*	This study
FCT1c	∆*fct1::FCT1-GFP-HYG*	This study
FCT2c	∆*fct2::FCT2-GFP-HYG*	This study
FCT1c-r	∆*fct1::FCT1-GFP-HYG; hH1::hH1-RFP-GEN*	mat1r × *fct1*
FCT2c-r	∆*fct2::FCT2-GFP-HYG; hH1::hH1-RFP-GEN*	mat1r × *fct2*
*fct1*-g	∆*fct1::GEN; GFP-HYG*	KM19 × *fct1*
*fct2*-g	∆*fct2::GEN; GFP-HYG*	KM19 × *fct2*
*fct1/2*-g	∆*fct1::GEN;* ∆*fct2::GEN; GFP-HYG*	KM19 × *fct1 fct2*

**Figure 1:**
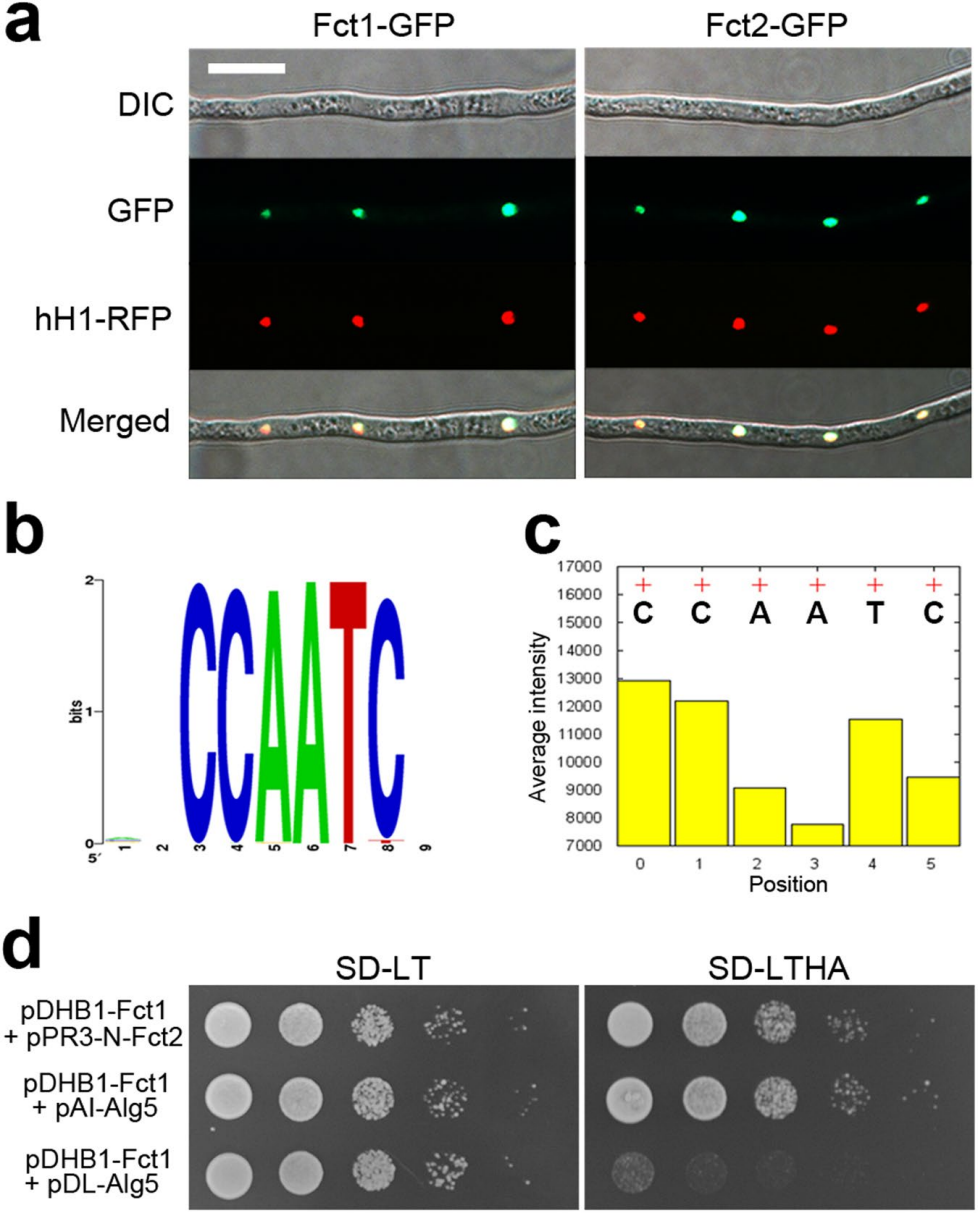
Characterization of the nuclear CCAAT-binding complex of *F. graminearum*. (**a**) Nuclear localization of Fct1-GFP and Fct2-GFP. FCT1c-r/FCT2c-r strains carrying both Fct1-GFP or Fct2-GFP and hH1-RFP were used for the colocalization study. Scale bar = 20 µm. (**b**) Consensus binding sequence identified via the PBM assay. Consensus sequences that robustly bound to the Fct2-DsRed fusion protein. (**c**) The effects of positional mutations in CCAATC. To visualize the effects of the mutations on the binding intensities in the consensus binding motif, the average binding intensities (+) of the probes containing the core consensus 6-mer binding motif CCAATC relative to those of probes with mutations at each position (bar) are plotted. (**d**) Yeast two-hybrid analysis of the interaction between Fct1 and Fct2. The plasmid pairs pDHB1-Fct1/pAI-Alg5 and pDHB1-Fct1/pDL2-Alg5 served as positive and negative controls, respectively. The growth of the transformed yeast was assayed on synthetic dextrose medium lacking Leu and Trp (SD-LT) or Leu, Trp, His, and Ade (SD-LTHA). The columns in each panel represent serial decimal dilutions.

We examined the complementation strains FCT1c and FCT2c, which harbour single copies of *FCT1-GFP* or *FCT2-GFP*, respectively, and observed GFP signal in the nuclei of both strains (Fig. 1a). To confirm the nuclear localization of Fct1-GFP and Fct2-GFP, FCT1c-r (*fct1*::*FCT1-GFP-HYG*; *hH1-RFP-GEN*) or FCT2c-r (*fct2*::*FCT2-GFP-HYG*; *hH1-RFP-GEN*) strains were generated via outcrosses between mat1r and FCT1c or FCT2c (Table 1). Both Fct1-GFP and Fct2-GFP colocalized with hH1-RFP and were highly fluorescent in all of the tested developmental stages, including the mycelia, indicating that Fct1 and Fct2 are constitutively expressed nuclear proteins.

### The CCAAT-binding protein Fct2 interacts with Fct1

Only two CCAAT-binding factor genes, *FCT1* and *FCT2*, are important for the development of the fungus *F. graminearum*, whereas the HAP complexes of higher eukaryotic organisms comprise three CCAAT-binding factors. To characterize the CCAAT-binding complex of *F. graminearum*, we first attempted to identify the DNA-binding sequence of Fct2 via a protein binding microarray (PBM) analysis (Fig. 1b,c). This PBM uses the target probes that are synthesized as quadruples of all possible 9-mer combinations, resulting in robust identification of the DNA-binding sequences of transcription factors^32^. The quadruple 9-mer (Q9)-based PBM analysis using the Fct2-DsRed fusion protein identified 4,526 putative DNA-binding sequences, with CCAATC as the predominant sequence (Fig. 1b). Individual substitutions at each position of the CCAATC sequence markedly reduced its DNA-binding affinity, suggesting that Fct2 has CCAAT DNA-binding activity and that it is a subunit of the CCAAT-binding complex in *F. graminearum* (Fig. 1c).

To identify the other components of the CCAAT-binding complex in *F. graminearum*, proteins that copurified with Fct2-GFP were analysed via mass spectrometry (Table 2). We successfully identified 13 putative Fct2-interacting proteins, and among seven CCAAT-binding factors, only Fct1 was revealed as an Fct2 interaction partner. To validate the physical interaction between Fct1 and Fct2, we used the DUALhunter yeast two-hybrid (Y2H) assay, because conventional Y2H systems cannot be used to analyse integral membrane proteins or transcription factors^33^. A strong positive interaction between Fct1 and Fct2 was indicated by yeast colony growth on medium lacking leucine (Leu), tryptophan (Trp), histidine (His), and adenine (Ade) (SD-LTHA) (Fig. 1d).

**Table 2:** Putative Fct2-interacting proteins identified via the affinity capture assay in *Fusarium graminearum*.

Locus ID	PSMs	Predicted function or gene name
FGRAMPH1_01G00849	10	Related to D-xylose reductase II,III protein
FGRAMPH1_01G03423	9	Related to 3-isopropylmalate dehydrogenase
FGRAMPH1_01G02933	9	Conserved hypothetical protein (*GzCCAAT002*/*FCT1*)
FGRAMPH1_01G19303	7	Probable ribosomal protein S25
FGRAMPH1_01G20773	6	Probable nucleolar protein NOP58
FGRAMPH1_01G12393	6	Related to endo-polygalacturonase 6
FGRAMPH1_01G11971	6	Phytoene dehydrogenase
FGRAMPH1_01G26385	5	Related to phosphomevalonate kinase
FGRAMPH1_01G02335	5	Probable cytochrome-b5 reductase
FGRAMPH1_01G27519	5	Probable hydroxymethylglutaryl-CoA synthase
FGRAMPH1_01G17119	5	Probable dead-box protein precursor CYT-19
FGRAMPH1_01G12125	5	Related to sedoheptulose-1, 7-bisphosphatase
FGRAMPH1_01G27113	5	Related to NIPSNAP protein

PSMs, total number of identified peptide sequences (peptide spectrum matches) for the protein.

**Figure 2:**
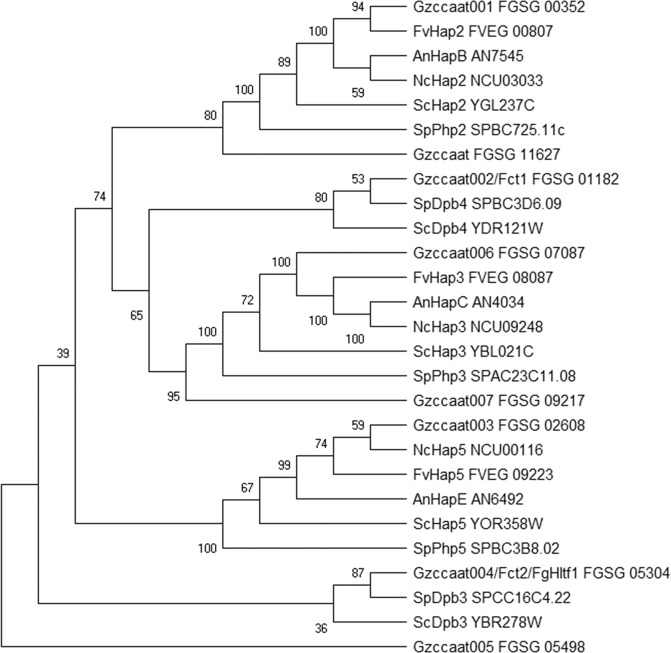
Phylogenetic tree of fungal CCAAT-binding factors. The alignment was performed with ClustalW, and MEGA X was used to perform a 1,000-bootstrap phylogenetic analysis using the neighbour joining method^62^. Bootstrap support is shown for each node.

Subsequently, phylogenetic analyses of the CCAAT-binding factors of *F*. *verticillioides*, *A. nidulans*, *Neurospora crassa*, *S. pombe*, and *S. cerevisiae* were performed (Fig. 2). Gzccaat001, Gzccaat006, and Gzccaat003 were identified as putative orthologues of Hap2, Hap3, and Hap5, respectively. Gzccaat007 and Gzccaat008 also grouped with Hap3 and Hap2, although they shared lower sequence similarity with the *S*. *cerevisiae* Hap3 and Hap2 (30.1 and 35.7% identity) than Gzccaat6 and Gzccaat001, respectively. Fct1 and Fct2 were shown to be evolutionarily closer to Dpb4 and Dpb3, respectively, than the Hap complex components. Dpb3 and Dpb4, small components of yeast DNA polymerase epsilon (Pol ε) subunits, each have a H2A/H2B histone-fold motif and bind to DNA sequence as a complex^20^.

### Fct1 and Fct2 are required for vegetative growth and perithecial development

**Figure 3:**
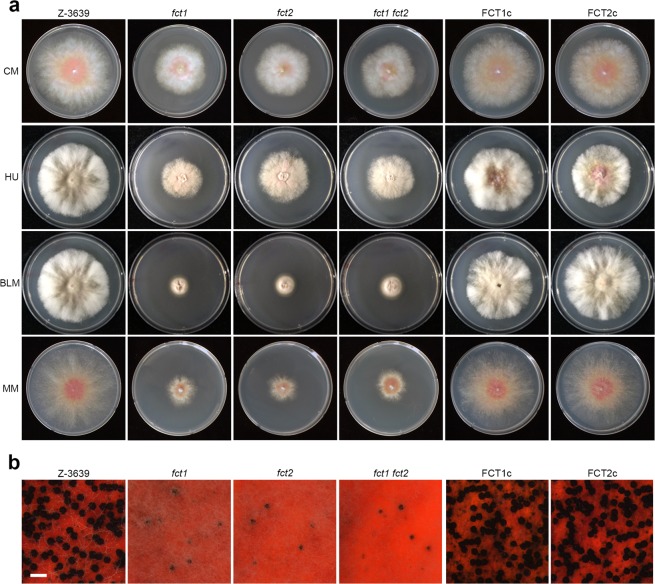
Vegetative growth and sexual development of *F. graminearum* strains. (**a**) The mycelial growth of *F. graminearum* strains on complete medium (CM), CM supplemented with 10 mM hydroxyurea (HU), 10 mU/ml bleomycin (BLM), and minimal medium (MM). The strains were imaged 5 days after inoculation. (**b**) Sexual development. A five-day-old culture on carrot agar medium was mock-fertilized to induce sexual reproduction, and the cultures were incubated for an additional 7 days. Scale bar = 500 µm.

To confirm the genetic requirement of *FCT1* and *FCT2* for vegetative growth and sexual development, we compared the phenotypes of the deletion mutants with the wild-type and complementation mutant strains. The deletion mutants grew poorly on complete medium (CM) and minimal medium (MM), whereas complementation fully rescued the growth defects (Fig. 3a). The increased sensitivity of the deletion mutants to the DNA damaging agents, hydroxyurea (HU) and bleomycin (BLM), was also rescued in the complemented strains. During sexual development, the *FCT1* and *FCT2* deletion mutants lost self-fertility, while the wild-type and complemented strains produced normal perithecia (Fig. 3b).

### *FCT1* and *FCT2* are important for virulence and total trichothecene production

**Figure 4:**
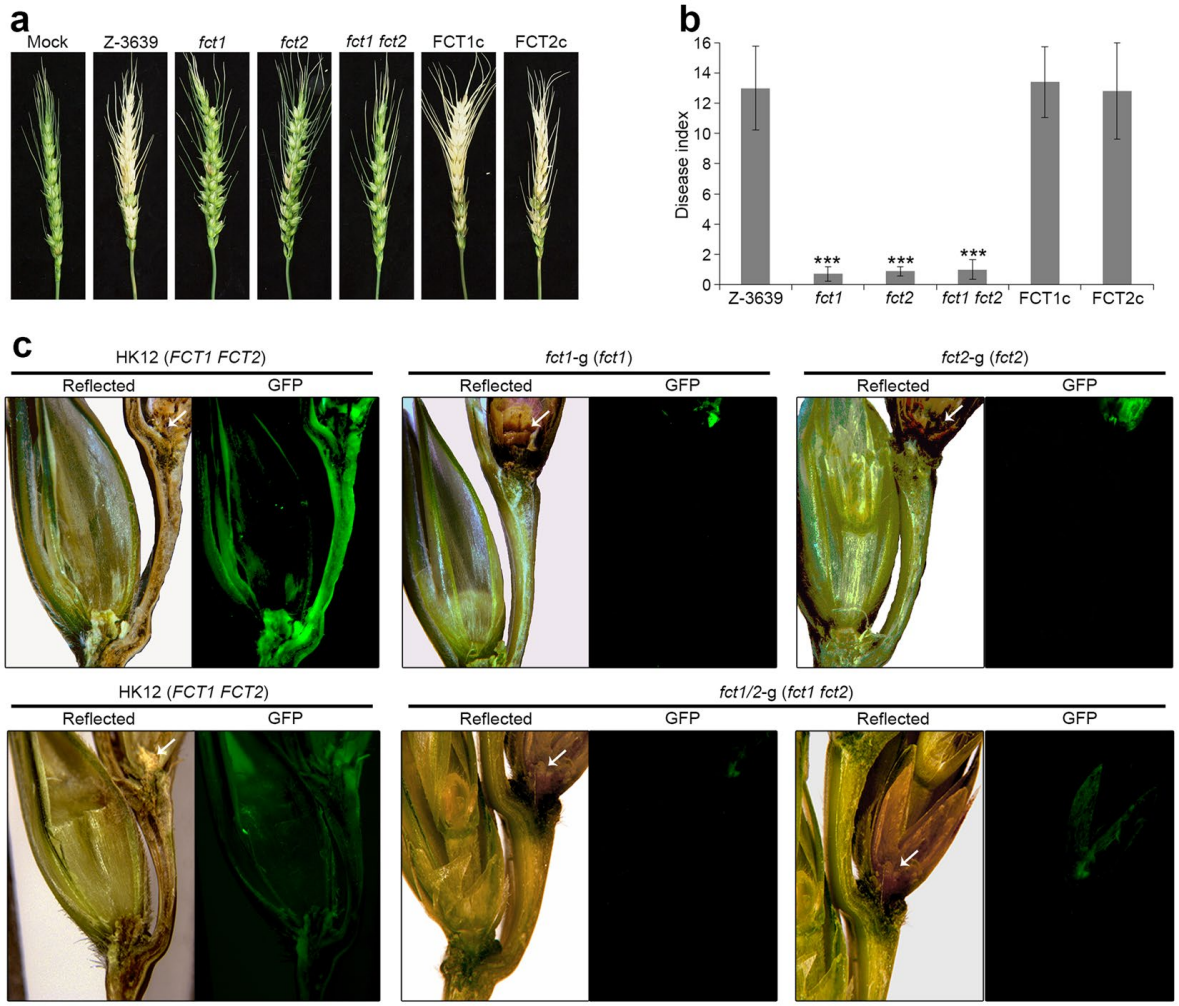
Virulence of *F. graminearum* strains. (**a**) Virulence on wheat heads. The centre spikelet of each wheat head was injected with 10 μl of a conidial suspension. Images were captured 21 days after inoculation. (**b**) Disease index. The disease index was estimated as the number of diseased spikelets on each wheat head. Asterisks represent significant difference between the wild-type strain and each mutant (p < 0.001). (**c**) Micrographs of manually generated sections after the infection of wheat. Wheat spikelets were inoculated with conidial suspensions from strains expressing GFP in the cytoplasm (HK12, *fct1*-g, *fct2*-g, and *fct1/2*-g). Infected wheat heads were longitudinally dissected 6 days after inoculation and examined under a fluorescence microscope. GFP fluorescence indicates hyphal spreading from the inoculation points. Arrowheads mark the inoculated spikelets. Reflected, reflected light.

To evaluate the involvement of *FCT1* and *FCT2* in virulence on flowering wheat heads, conidial suspensions of strains were point-inoculated on a spikelet, and the plants were incubated in a greenhouse. The wild-type strain induced normal head blight symptoms, manifesting as discoloration at 21 days after inoculation, whereas the *fct1* and *fct2* strains were restricted to the initial infection sites and were unable to spread to adjacent spikelets on the head (Fig. 4a,b).

To further visualize the spread of the mycelia on wheat heads during infection, the strains *fct1*-g (*∆fpo1::GEN; GFP-HYG*) and *fct2*-g (*∆fpo2::GEN; GFP-HYG*) were generated via outcrossing of KM19^34^ with the *fct1* or *fct2* deletion mutants (Table 1). By 6 days after inoculation, hyphae of the HK12 strain (which carries wild-type *FCT*s and expresses cytosolic GFP) had spread to adjacent spikelets through rachis nodes (Fig. 4c). However, fluorescent hyphae of the *fct1*-g and *fct2*-g strains were only detected on the inoculated spikelets and failed to penetrate rachis nodes.

**Figure 5:**
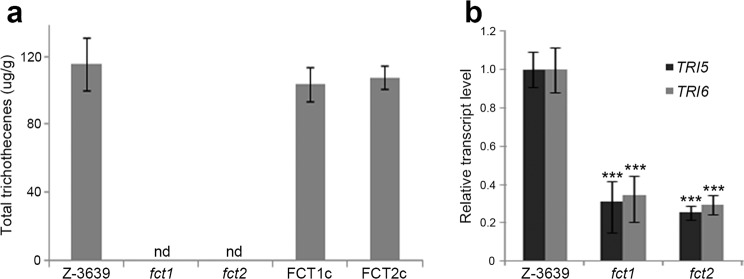
Trichothecene production by the *F. graminearum* strains. (**a**) Total trichothecene production. Each strain was grown in minimal medium containing 5 mM agmatine (MMA) for 7 days. Trichothecenes were analysed via gas chromatography-mass spectrometry (GC-MS) and quantified based on the biomass of each strain. (**b**) Transcript levels of *TRI5* and *TRI6* in the *F. graminearum* strains. The transcript levels were analysed via quantitative real-time PCR (qRT-PCR) 4 days after inoculation in MMA. Asterisks represent significant differences in the relative transcript levels of *TRI5* and *TRI6* between the wild-type strain and each mutant (p < 0.001).

The levels of trichothecene synthesized by both the *fct1* and *fct2* deletion mutants were mostly undetectable, whereas the wild-type and complementation strains accumulated high amounts of trichothecenes (Fig. 5a). Furthermore, the transcriptional levels of the trichothecene biosynthetic genes *TRI5* and *TRI6* were also significantly reduced in the deletion mutants (Fig. 5b).

### Phenotypes of double mutant revealed genetic interaction of *FCT1* and *FCT2*

We hypothesized that if Fct1-Fct2 complex formation is crucial for the biological functions of these two proteins, then the phenotypic defects of the *fct1* and *fct2* single deletion mutants should be similar to those of an *fct1 fct2* double deletion mutant. We generated *fct1 fct2* double deletion mutants by deleting the *FCT2* gene in an *fct1* mutant (Supplementary Fig. S2), and the *fct1 fct2* double deletion mutant showed indistinguishable phenotypes from those of the single deletion mutants (*fct1* and *fct2*) under our tested assay conditions.

## Discussion

In this study, we identified and characterized the CCAAT-binding transcription factors in the plant pathogenic fungus *F. graminearum*. Our biochemical, cytological, and genetic results demonstrated that the CCAAT-binding complex of *F. graminearum* comprises only two CCAAT-binding factors (Fct1 and Fct2) that are not highly conserved in other fungi or higher eukaryotes. Moreover, in-depth phenotypic analysis revealed that the Fct1-Fct2 complex is involved in various biological processes in *F. graminearum*, including vegetative growth, sexual reproduction, virulence, and mycotoxin production.

In most eukaryotic organisms, the heme activator protein (HAP), also known as nuclear factor Y (NF-Y) or CCAAT-binding factor (CBF), is composed of three subunits: Hap2 (also termed NF-YA, CBF-B, or HapB), Hap3 (NF-YB, CBF-A, or HapC), and Hap5 (NF-YC, CBF-C, or HapE), indicating that this complex structure is evolutionally conserved in eukaryotes^35–37^. Because each of these subunits is required for the DNA-binding activity of the complex, they are all essential for the function of the CCAAT-binding complex^38–40^. Biochemical studies of the interactions between these subunits has revealed that Hap3 and Hap5 form a tight dimer via a protein-protein interaction that is similar to the head to tail association of histones H2A/H2B and that this dimer offers a complex binding surface for Hap2^39,41^. Interestingly, although the trimeric complex is sufficient to regulate gene expression in mammalian cells, orthologues of an additional component, Hap4 (HapX) or Hap4-like proteins, have been identified in fungi, including *S*. *cerevisiae*, *S*. *pombe*, *Aspergillus*, and *Candida* species^3,42–44^. Hap4 orthologues harbour a bZIP domain and a conserved 16-amino acid motif that is required for its interaction with the Hap2/Hap3/Hap5 complex. The Hap2, Hap3, and Hap5 complex assembles in the cytoplasm and is then transported to the nucleus via the nuclear localization signal (NLS) of Hap2^11,45,46^. Subsequently, the heterotrimer complex binds to the promoters of target genes containing a CCAAT sequence and recruits Hap4 for subsequent gene activation^46^.

A histone fold is a structurally conserved motif identified near the C-terminus in core histones and is responsible for the ability of histones to bind and form heterodimers^47^. In addition to core histones, a similar secondary structure arrangement has been observed in several nuclear proteins involved in DNA metabolism, including TATA box-binding protein-associated factors, the DNA polymerase II subunits Dpb3 and Dpb4, and the CCAAT-binding complex proteins Hap3 (NF-YB) and Hap5 (NF-YC). The structure of the NF-YC-NF-YB complex resembles that of the H2A-H2B histone dimer^48^. Crystal structure analysis of the *A*. *nidulans* HAP complex showed that the complex specifically recognizes the CCAAT box of the promoter, and the *S*. *cerevisiae* Dpb3-Dpb4 heterodimer associates with double-stranded DNA without a preference for specific DNA sequences^18,49^. In this study, based on phylogenetic analysis, we determined that Fct1 and Fct2 are more closely related to Dpb4 and Dpb3, respectively, than to Hap complex components and that the two proteins interact with each other. In addition, protein binding microarray results showed that Fct2 specifically bound to the CCAAT sequence, unlike the Dpb3-Dpb4 complex, which binds to nonspecific double-stranded DNA. The deletion of *FCT1* and *FCT2*, alone or in combination, resulted in an increased sensitivity of *F. graminearum* to DNA damaging agents. We speculate that Fct1 and Fct2 may be responsible for histone modification and DNA replication, although further investigation is needed to confirm this hypothesis.

In *F. graminearum*, among eight transcription factors identified as CCAAT-binding factors, Gzccaat001 (FGSG_00352), Gzccaat006 (FGSG_07087), and Gzccaat003 (FGSG_02608) are orthologous proteins of Hap2, Hap3, and Hap5, respectively (Fig. 2). However, deletion of these genes did not significantly affect *F*. *graminearum* phenotypes, such as mycelial growth, sexual development, toxin production, and virulence^29^. Thus, further in-depth genetic and biochemical analyses of these genes are needed to reveal the biological function of the HAP complex in *F*. *graminearum*.

Members of the CCAAT-binding factor gene family have diverse roles as transcriptional regulators for multiple cellular processes, including cell proliferation, apoptosis, differentiation, the control of metabolic pathways, the establishment of cell fate and identity, and stress responses in animals and plants^35,50,51^. In the yeast *S. cerevisiae*, CCAAT boxes are present in the promoters of cytochrome-encoding genes and in other genes involved in the use of nonfermentable carbon sources^38^ and in nitrogen metabolism^52^. CCAAT boxes are present in the promoters of genes involved in penicillin biosynthesis, and the HAP complex is also involved in the metabolism of carbon and nitrogen in the filamentous fungus *A. nidulans*^40^. Although *F. graminearum* has a simple CCAAT-binding complex structure compared to those of other eukaryotes, its Fct1-Fct2 complex has evolved to possess important functions in the development and virulence of *F. graminearum*.

In summary, in this study, we report that Fct1 and Fct2 are distinct components of the CCAAT-binding complex that have histone-fold motifs and are involved in various fungal developmental processes and virulence in *F. graminearum*. To the best of our knowledge, this is the first study on transcription factors containing CCAAT-binding domains in a plant pathogenic fungus, the results of which provide important information on the molecular mechanisms underlying the functions of CCAAT-binding factors in eukaryotes. Compared with what is known regarding the structure and function of the HAP complexes of model organisms, our knowledge on the CCAAT-binding complexes of plant pathogenic fungi, including *F. graminearum*, is lacking. Therefore, further studies will be needed to characterize the target genes and specialized functions of the CCAAT-binding complex in *F. graminearum*.

## Methods

### Fungal strains and media

All strains used in this study are listed in Table 1. The *F*. *graminearum* wild-type strain Z-3639^53^ and mutants derived from this strain were maintained according to the *Fusarium* laboratory manual^54^. A transgenic strain, mat1r^55^, harbouring both a *MAT1-1* deletion and red fluorescent protein (RFP)-tagged histone H1 was used in the colocalization study. Minimal liquid medium supplemented with 5 mM agmatine (MMA) was used for the trichothecenes analysis^56^.

### Genetic manipulations, primers, and sequencing

Fungal genomic DNA was extracted according to the *Fusarium* laboratory manual^54^. Total RNA was isolated from mycelia ground in liquid nitrogen using an Easy-Spin Total RNA Extraction kit (Intron Biotech, Seongnam, Republic of Korea). Standard protocols were followed for restriction endonuclease digestion, agarose gel electrophoresis, and DNA gel blot hybridization with ^32^P labelled probes^57^. The PCR primers used in this study were synthesized at an oligonucleotide synthesis facility (Bionics, Seoul, Republic of Korea) (Supplementary Table S1).

### Targeted deletion and complementation

The double-joint (DJ) PCR strategy was used to construct the fusion PCR products required to generate the targeted gene deletion and complementation^58^ via homologous recombination. The open reading frames (ORFs) of *FCT1* or *FCT2* in the *F. graminearum* wild-type strain Z-3639 were replaced with the geneticin resistance gene^31^ to create the deletion mutants. The *fct1* and *fct2* double mutant was generated by replacement of *FCT2* gene with the hygromycin resistance gene (*HYG*) in the *fct1* mutant. For the complementation and cellular localization assays, the 5′ flanking region, including the ORF with its own promoter, and the 3′ flanking region were amplified from genomic DNA of the wild-type strain using the primer pairs FCT1-5F/FCT1-5R GFP and FCT1-3F GFP/FCT1-3R, respectively. The *GFP-HYG* construct was amplified from the plasmid pIGPAPA using the primers pIGPAPA-sGFP/HYG-F1. The three amplicons were then fused via a second round of DJ PCR, after which the fusion constructs for transformation were amplified with nested primers using the second round PCR product as a template. Fungal transformation was performed as previously described^55^. The FCT2c strain, which was used to investigate cellular localization, was generated via the same strategy.

### Microscopic observation

Microscopic observations were performed using a DE/Axio Imager A1 microscope (Carl Zeiss, Oberkochen, Germany) with the filter set 38HE (excitation 470/40; emission 525/50) for GFP and the filter set 15 (excitation 546/12; emission 590) for RFP.

Wheat heads inoculated with the GFP-expressing strains were observed as previously described^34^. Infected wheat heads were longitudinally dissected 6 days after inoculation and examined under a fluorescence microscope. Longitudinal sections cut through the centre of the spikelets were prepared freehand using a clean scalpel. The sectioned wheat heads were observed under reflected light and GFP fluorescence light (470 nm excitation and 525 nm emission wavelength filters) on a SteREO Lumar V12 microscope (Carl Zeiss).

### Protein binding microarray (PBM) analysis

To determine the DNA-binding sequence of Fct2, a protein binding microarray assay was performed as previously described^32,59^. The full-length cDNA of *FCT2* was inserted into the pET-DsRed expression vector to generate pET-Fct2Red, and the Fct2-DsRed fusion protein was expressed in the *E. coli* strain BL21-ColonPlus. Subsequently, the purified protein was incubated with a Q9 protein-binding microarray (Q9-PBM), which includes 232,145 quadruple probes, including 131,072 probes for all possible 9-mers, each of which was concatenated four times. Fluorescence images were captured using a GenePix 4000B microarray scanner (Molecular Devices, San Jose, CA, USA). The consensus binding sequence was determined based on the fluorescence signal intensities according to previously described methods^32^.

### Affinity purification and mass spectrometry analysis

To capture Fct2-interacting proteins, cell lysates prepared from two independent FCT2c strains were incubated with magnetic beads conjugated to a mouse anti-GFP antibody (MBL International, Woburn, MA, USA) following the manufacturer’s instructions. After incubating at 4 °C for overnight, the magnetic beads were washed six times with phosphate-buffered saline (PBS; 137 mM NaCl, 2.7 mM KCl, 10 mM Na_2_HPO_4_, and 1.8 mM KH_2_PO_4_, pH 7.4) prior to resolving the proteins via SDS-PAGE (12%). The proteins separated via SDS-PAGE were digested with trypsin *in situ* and then analysed using a Q Exactive™ nano high resolution LC/MS MS spectrometer (Thermo Scientific, Waltham, MA, USA). The resulting peptide amino acid sequences from the LC-MS/MS data were identified in sequences in the *Fusarium graminearum* database (https://fungidb.org/)^60^. The results obtained for the wild-type strain were used as a negative control.

### Yeast two-hybrid assay (Y2H)

The Y2H assay was conducted using a DUALhunter kit (Dualsystems Biotech, Zurich, Switzerland) following the manufacturer’s instructions. To obtain cDNA, total RNA extracted from fungal cultures grown on carrot agar medium was reverse transcribed with SuperScriptIII reverse transcriptase (Invitrogen, Carlsbad, CA, USA). Each ORF was PCR amplified using primers with a SfiI restriction site (Supplementary Table S1). The full cDNAs of *FCT1* and *FCT2* were cloned into pDHB1, a Cub-based bait vector, and pRN3-N, a NubG-based prey vector, respectively (Dualsystems Biotech). After cotransformation of these vectors into *S. cerevisiae* NMY51 (*MAT a his3*Δ*200 trp1-901 leu2-3, 112 ade2 LYS2::*(*lexApo*)*4- HIS3 ura3*::(*lexApo*)*8-lacZ ade2*::(*lexApo*)*8-ADE2 GAL4*), a colony picked from the SD-Leu-Trp plates was grown in liquid SD-Leu-Trp medium, and the resulting cells were then spotted onto selective plates (SD-Leu-Trp-His-Ade). Strains carrying the empty vectors and pDL-Alg5 (−) were included as negative controls, while pAl-Alg5 (+) was included as a positive control.

### Sexual crosses

Aerial mycelia were removed from cultures grown on carrot agar medium for 5 days with 0.5 ml of a 2.5% Tween 60 solution to induce sexual reproduction. The plates were incubated under a near-UV light (wavelength: 365 nm; Sankyo Denki Co., Ltd., Tokyo, Japan) at 25 °C for 7 to 10 days. For the outcrosses, mycelia from a female strain grown on carrot agar medium were fertilized with 1 ml of conidial suspension (10^6^ conidia/ml) obtained from a male strain.

### Virulence test and trichothecene analysis

For the virulence test, the point inoculation method was performed as previously described^55^. Conidial suspensions (10^5^ conidia/ml) were prepared for each strain, and 10 μl of each suspension was injected into the centre spikelet of a wheat head (cultivar: Eunpamil). After inoculation, the wheat plants were incubated in a humidified chamber for 3 days and then transferred to a greenhouse. Spikelets exhibiting disease symptoms were counted 21 days after inoculation. The experiment was performed with five replicate inoculations per strain, and two independent mutant strains were used for the experiment.

Trichothecene analysis was performed as previously described^29^. Briefly, MMA cultures were extracted with ethyl acetate, and the extracts were concentrated to dryness. A portion of each extract was derivatized with Sylon BZT (BSA + TMCS + TMSI, 3:2:3 respectively, Supelco, Bellefonte, PA, USA) and analysed with a Shimadzu QP-5000 gas chromatograph mass spectrometer (GC-MS, Shimadzu, Kyoto, Japan) using the relevant ion-monitoring mode as previously described^61^. The trichothecenes were quantified based on the biomasses produced by each strain, and the experiment was repeated three times.

### Quantitative real time (qRT)-PCR

Total RNA was prepared using an Easy-Spin Total RNA Extraction kit (Intron Biotech). The first strand cDNA was synthesized with SuperScriptIII reverse transcriptase (Invitrogen). Quantitative real-time PCR (qRT-PCR) was performed using SYBR Green Supermix (Bio-Rad, Hercules, CA, USA) and a 7500 real-time PCR system (Applied Biosystems, Foster City, CA, USA) with the corresponding primers (Supplementary Table S1). The endogenous housekeeping gene cyclophilin (*CYP1*) was used as an endogenous control for normalization. The qRT-PCR assay was repeated three times with three replicates per run, and the transcript levels relative to that of the housekeeping gene were expressed as 2^−ΔΔCT^^31^.

## Supplementary information

Supplementary information.
