# Supplementary material for: Characterization of the CCAAT-binding transcription factor complex in the plant pathogenic fungus Fusarium graminearum
Source: Sci Rep. 2020 Mar 17;10:4898. doi: 10.1038/s41598-020-61885-4 (PMC7078317; doi:10.1038/s41598-020-61885-4)
Supplement: Supplementary file 1 — Supplementary information. [file 41598_2020_61885_MOESM1_ESM.docx]

**Supplementary information**

**Characterization of the CCAAT-binding transcription factor complex in the plant pathogenic fungus *Fusarium graminearum***

**Jung-Eun Kim^1^, Hyejin Nam^1^, Jiyeun Park^1^, Gyung Ja Choi^2^, Yin-Won Lee^1^ & Hokyoung Son^1^**

^1^Research Institute of Agriculture and Life Sciences and Department of Agricultural Biotechnology, Seoul National University, 08826, Seoul, Republic of Korea. ^2^Eco-friendly New Materials Research Group, Research Center for Biobased Chemistry, Division of Convergence Chemistry, Korea Research Institute of Chemical Technology, Daejeon 34114, Republic of Korea. Jung-Eun Kim and Hyejin Nam contributed equally. Correspondence and requests for materials should be addressed to H.S. (email: hogongi7@snu.ac.kr).

**Table S1.** Primers used in this study.

| **Primer** | **Sequence (5′ → 3′)** | **Description** |
| --- | --- | --- |
| FCT1-5F | GGGAGGCCTTTCACCACGAC | Forward and reverse primer for amplification of the 5′ flanking region of *FCT1* with a tail for geneticin resistance gene cassette fusion |
| FCT1-5R | gcacaggtacacttgtttagagCTTTTGCGCGAGCTGGATTTAC |  |
| FCT1-3F | ccttcaatatcatcttctgtcgAGGAAGGAAAGGAAGACAGAGACGAG | Forward and reverse primer for amplification of the 3′ flanking region of *FCT1* with a tail for geneticin resistance gene cassette fusion |
| FCT1-3R | TCACCAACACAGTCGAACATCAGG |  |
| FCT1-5N | GAGCATGCGTACCCCAGTATCCAT | Forward and reverse nest primers for third fusion PCR for amplification of the construct for *FCT1* deletion |
| FCT1-3N | GGTGGTGATACGAAATGACGGAATG |  |
| FCT1-5F GFP | = FCT1-5F | Forward and reverse primer for amplification of the 5′ flanking region of *FCT1* with a tail for GFP tagging complementation |
| FCT1-5R GFP | gaacagctcctcgcccttgctcacATCGCTCTCATCCCCATCCAGAGC |  |
| FCT1-3F GFP | cctccactagctccagccaagccTGCAGAGCAGAATCAAGCCATT | Forward and reverse primer for amplification of the 3′ flanking region of *FCT1* with a tail for GFP tagging complementation |
| FCT1-3R GFP | = FCT1-3R |  |
| FCT1-5N GFP | = FCT1-5N | Forward and reverse nest primers for third fusion PCR for amplification of the construct for GFP tagging complementation |
| FCT1-3N GFP | = FCT1-3N |  |
| FCT3-5F | GCTGGCGCAATGGGGAAAAA | Forward and reverse primer for amplification of the 5′ flanking region of *FCT3* with a tail for geneticin resistance gene cassette fusion |
| FCT3-5R GFP | gcacaggtacacttgtttagagTAGTGTTGTAAGGCATGGTGGTTGAT |  |
| FCT3-3F GFP | ccttcaatatcatcttctgtcgAAAATCACCAGAGACGCCAGAGCA | Forward and reverse primer for amplification of the 3′ flanking region of *FCT3* with a tail for geneticin resistance gene cassette fusion |
| FCT3-3R | TGGATGTGGCGTTGTTGGAGTT |  |
| FCT3-5N | TGTACCATTCCCTTCTCCCCAACAG | Forward and reverse nest primers for third fusion PCR for amplification of the construct for *FCT3* deletion |
| FCT3-3N | TTATCGAGAAACCAAGAGCAATCACTC |  |
| FCT3-5F GFP | = FCT3-5F | Forward and reverse primer for amplification of the 5′ flanking region of *FCT3* with a tail for GFP tagging complementation |
| FCT3-5R GFP | gaacagctcctcgcccttgctcacGCCTGACATGGTCACATCTC |  |
| FCT3-3F GFP | cctccactagctccagccaagccTTACAAGTCGTAATGAACAGTGAC | Forward and reverse primer for amplification of the 3′ flanking region of *FCT3* with a tail for GFP tagging complementation |
| FCT3-3R GFP | = FCT3-3R |  |
| FCT3-5N GFP | = FCT3-5N | Forward and reverse nest primers for third fusion PCR for amplification of the construct for GFP tagging complementation |
| FCT3-3N GFP | = FCT3-3N |  |
| Gen-for | CGACAGAAGATGATATTGAAGG | Forward and reverse primers for amplification of the geneticin cassette from the pII99 vector |
| Gen-rev | CTCTAAACAAGTGTACCTGTG |  |
| pIGPAPA-sGFP F | GTGAGCAAGGGCGAGGAGCTG | Forward and reverse primers for amplification of the *GFP-HYG* construct from pIGPAPA vector |
| HYG-F1 | GGCTTGGCTGGAGCTAGTGGAGG |  |
| FCT1-bait-F | aacgcagagaggccattacggccATGGACAATGTGTCACCATTG | For Yeast two Hybrid assay |
| FCT1-bait-R | aacgcagagaggccgaggcggccaaATCGCTCTCATCCCCATCC |  |
| FCT3-bait-F | aacgcagagaggccattacggccATGCCTTACAACACTACAGCTATTC |  |
| FCT3-bait-R | aacgcagagaggccgaggcggccgCTAGCCTGACATGGTCACATCT |  |
| FCT1-RT-F | GGAAAAGGAGAAGGATAAAGAGCATAAG | For realtime-PCR of *FCT1* |
| FCT1-RT-R | CCAGCGTTCACAGTGTTTTCGT |  |
| FCT3-RT-F | GATCGCCCTCTGCTCCAACA | For realtime-PCR of *FCT3* |
| FCT3-RT-R | TGATGTGCGACAGCAGAGGC |  |
| TRI5-RT-F | GCCATTTTGGACCTTTCTGCTCATT | For realtime-PCR of *TRI5* |
| TRI5-RT-R | GCCATAGAGAAGCCCCAACACAAT |  |
| TRI6-RT-F | GGCAACCATTCAAGCGCTTTTTCT | For realtime-PCR of *TRI6* |
| TRI6-RT-R | CACCCTGCTAAAGACCCTCAGACATT |  |
| CYP1-RT-F | TCAAGCTCAAGCACACCAAGAAGG | For realtime-PCR of *CYP1* (reference gene) |
| CYP1-RT-R | GGTCCGCCGCTCCAGTCT |  |


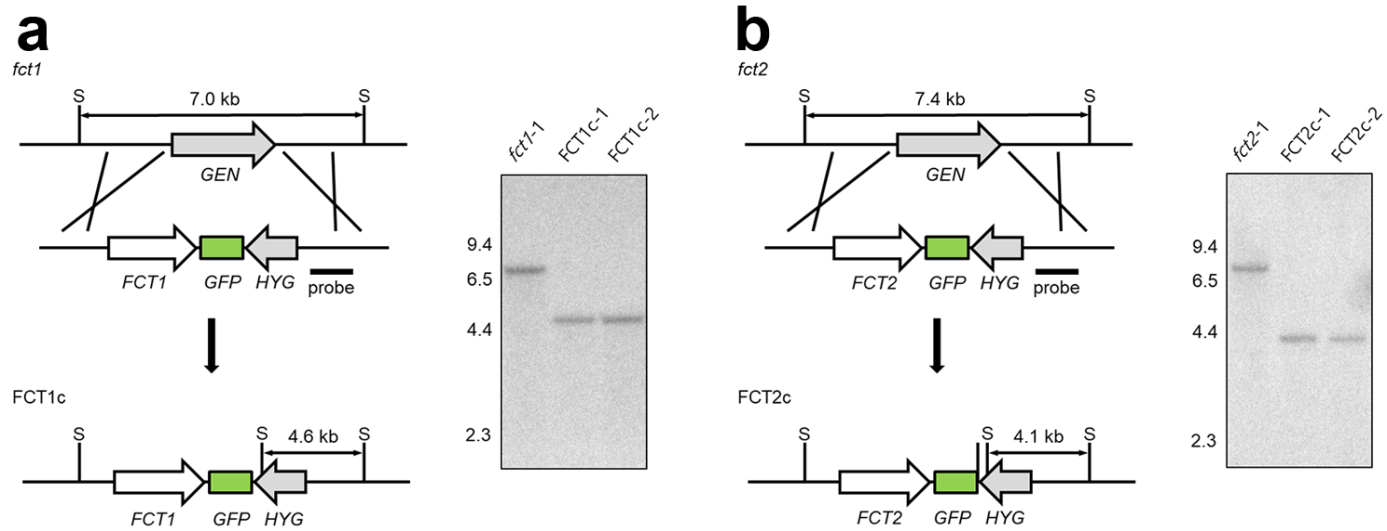


**Figure S1. Genetic complementation.** Genetic complementation of *fct1* (**a**) and *fct2* (**b**). Schematic illustrating the strategy for genetic complementation of *fct1* or *fct2* (left panel). Southern blot analyses confirming genetic manipulations (right panel). Lane 1, deletion mutant; Lane 2 and 3, complementation mutants. Sizes of the DNA standards (kb) are indicated to the left of the blot. S, SalⅠ.


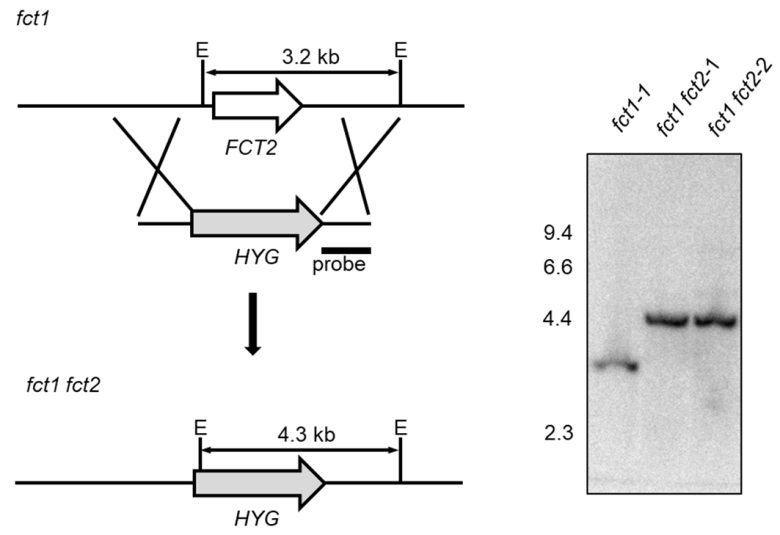


**Figure S2. Double deletion of *FCT1* and *FCT2*.** Schematic illustrating the strategy for deltion of *FCT2* (left panel) in the *fct1* deletion mutant. Southern blot analyses confirming genetic manipulations (right panel). Lane 1, deletion mutant; Lane 2 and 3, complementation mutants. Sizes of the DNA standards (kb) are indicated to the left of the blot. E, EcoRⅠ.
